# Printed 2 V-operating organic inverter arrays employing a small-molecule/polymer blend

**DOI:** 10.1038/srep34723

**Published:** 2016-10-04

**Authors:** Rei Shiwaku, Yasunori Takeda, Takashi Fukuda, Kenjiro Fukuda, Hiroyuki Matsui, Daisuke Kumaki, Shizuo Tokito

**Affiliations:** 1 Research Center for Organic Electronics (ROEL), Graduate School of Science and Engineering, Yamagata University, 4-3-16 Jonan, Yonezawa, Yamagata 992-8510, Japan; 2 Functional Polymers Research Laboratory, Tosoh Corporation, 1-8, Kasumi, Yokkaichi, Mie 510-8540, Japan; 3 Japan Science and Technology Agency, PRESTO,4-1-8, Honcho, Kawaguchi, Saitama 332-0012, Japan

## Abstract

Printed organic thin-film transistors (OTFTs) are well suited for low-cost electronic applications, such as radio frequency identification (RFID) tags and sensors. Achieving both high carrier mobility and uniform electrical characteristics in printed OTFT devices is essential in these applications. Here, we report on printed high-performance OTFTs and circuits using silver nanoparticle inks for the source/drain electrodes and a blend of dithieno[2,3-d;2′,3′-d′]benzo[1,2-b;4,5-b′]dithiophene (DTBDT-C_6_) and polystyrene for the organic semiconducting layer. A high saturation region mobility of 1.0 cm^2^ V^−1^ s^−1^ at low operation voltage of −5 V was obtained for relatively short channel lengths of 9 μm. All fifteen of the printed pseudo-CMOS inverter circuits were formed on a common substrate and operated at low operation voltage of 2 V with the total variation in threshold voltage of 0.35 V. Consequently, the printed OTFT devices can be used in more complex integrated circuit applications requiring low manufacturing cost over large areas.

As a result of their printability and potential in realizing low-cost and large-area electronic devices, organic thin-film transistors (OTFTs) have attracted significant attention in the research and development of next-generation thin-film electronics^1^,^2^. Among the various kinds of printing technologies that are typically used, non-contact printing methods such as ink-jet printing and dispenser printing provide for drop-on-demand fabrication from digital data and can directly pattern customizable elements on a substrate. Moreover, OTFTs have intrinsic mechanical flexibility due to the loose van der Waals force between organic molecules. Furthermore, OTFTs can be formed on very thin plastic films by low temperature processes, which gives the devices excellent mechanical flexibility due to the low bending-induced strain^3^. These features are ideal for low-cost and flexible electronic applications, such as radio-frequency identification (RFID) tags^4^, medical sensors^5^, and biosensors^6^,^7^.

To realize OTFT devices for use in practical device and circuit applications, achieving both high field-effect mobility and uniform electrical characteristics is essential but also challenging. Although small-molecule semiconductor TFT devices possess high carrier mobility, they also exhibit large variations in electrical characteristics^8^. On the other hand, OTFTs based on polymeric semiconductor materials show more uniform electrical performance with lower carrier mobility^9^. When fabricating practical OTFTs using printing technology, we face this trade-off between high mobility and uniform electrical performance, although a compromise should be found.

Towards satisfying these requirements, improving the semiconductor layer formation properties with techniques that employ a blend of a small-molecule semiconductor and insulating polymers have recently been developed^10^,^11^. By using the solution that contains a small-molecule semiconductor and insulating polymers for the organic semiconducting layers, the morphology of the semiconductor films is changed as a result of the vertical nanophase separation phenomenon, which can lead to the improvement in carrier mobility and reduced variation in electrical characteristics^12^. These reports were based on the use of evaporated metals for the source/drain electrodes. TFT device performance achieved by applying a blended semiconductor solution as well as printed metal electrodes has yet to be evaluated.

Recently, we reported on the printed OTFTs with uniform electrical characteristics using a small-molecule semiconductor material named dithieno[2,3-*d*;2′,3′-*d*′]benzo[1,2-*b*;4,5-*b*′]dithiophene (DTBDT-C_6_)^13^,^14^ and described its potential in printed OTFT devices^15^. The OTFT devices using printed source/drain electrodes had high contact resistance (*R*_C_) because the thickness uniformity of printed electrodes was generally worse than that of evaporated electrodes due to the coffee ring effect, which causes a non-uniform ring-like profile^16^. Moreover, the work function and conductivity of printed electrodes deviated from their bulk values because of impurities^17^, which can be the origin of the poor charge transport from the electrodes to a semiconducting layer. Therefore, forming OTFT devices using printed electrodes with short channel lengths (<10 μm) and low-voltage operation is difficult.

In this study, we report on high performance organic transistors with inkjet-printed silver electrodes and the channel of DTBDT-C_6_/polystyrene blend. Blending small molecular semiconductors and polymer insulators reduced the contact resistance at the printed electrodes significantly, which enables us to realize high mobility up to 1.0 cm^2^ V^−1^ s^−1^ at a short channel length of 9 μm. Blending also improves the uniformity of the device characteristics. Their potential for practical integrated circuits were evaluated by fifteen pseudo-complementary metal-oxide-semiconductor (pseudo-CMOS) type logic inverters on the same substrate. All fifteen inverters worked properly at operation voltage of 2 V owing to the quite uniform threshold voltages of the OTFTs.

## Results and Discussion

### Electrical characteristics of small-molecule semiconductor/insulating polymer blend TFT devices

**Figure 1:**
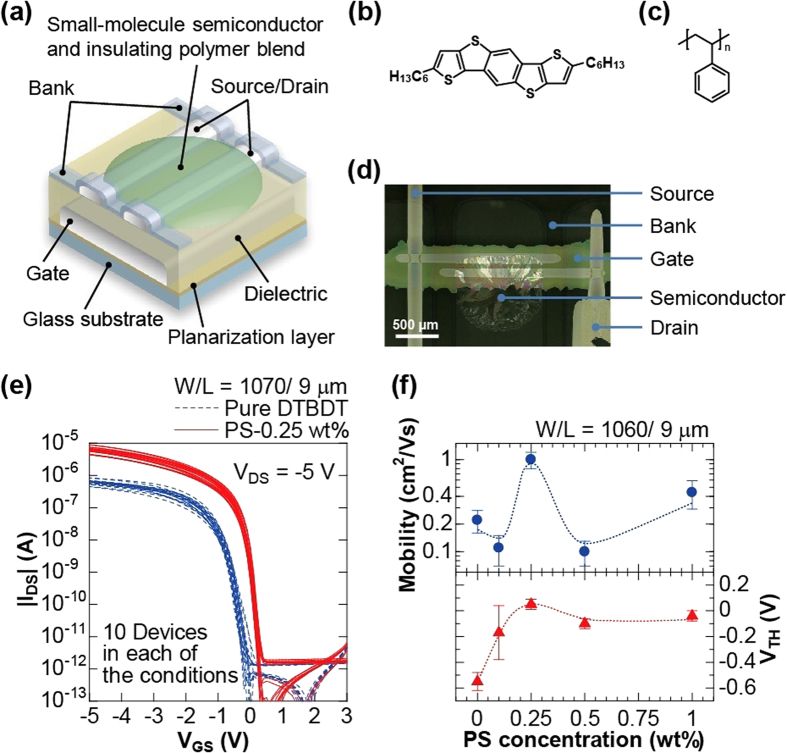
Printed small-molecule/polymer blend TFTs. (**a**) Schematic illustration of the printed organic TFT devices. Chemical structure of (**b**) DTBDT-C_6_ and (**c**) Polystyrene (PS). (**d**) Photograph of a fabricated organic TFT device. (**e**) Transfer characteristics of the fabricated OTFTs. Plotted are drain-source current (*I*_DS_) for pure DTBDT-C_6_ devices (dashed blue lines) and 0.25 wt%-PS-blended DTBDT-C_6_ devices (solid red lines), as a function of gate-source voltage (*V*_GS_) and at a drain-source voltage (*V*_DS_) of −5 V. The average mobility in the saturation region were 0.22 cm^2^ V^−1^ s^−1^ with pure DTBDT-C_6_ and 1.00 cm^2^ V^−1^ s^−1^ with 0.25 wt%-PS-blended DTBDT-C_6_. (**f**) Dependency of the mobility (blue circles) and threshold voltage (*V*_TH,_ red triangle) on PS concentration.

Figure 1a shows a schematic illustration of the fabricated bottom-gate, bottom-contact OTFT device. All layers except the dielectric layer were formed by using printing methods at a maximum process temperature of 140 °C. Two silver nanoparticle inks were used for the electrodes whose formulations were previously reported in the literature^18^,^19^. For the organic semiconducting layers, we employed a blend of DTBDT-C_6_ (1 wt%, Fig. 1b) and polystyrene (0, 0.1, 0.25, 0.5, and 1.0 wt%, Fig. 1c) in toluene. Figure 1d shows a photograph of a fabricated OTFT device. A printed fluoropolymer layer was used as a confining bank layer, whereby the semiconducting layer was printed in the area defined by the bank layer using dispenser equipment. These device fabrication processes used non-contact and plateless printing techniques such as ink-jet printing and dispenser printing for highly customizable patterning.

**Figure 2:**
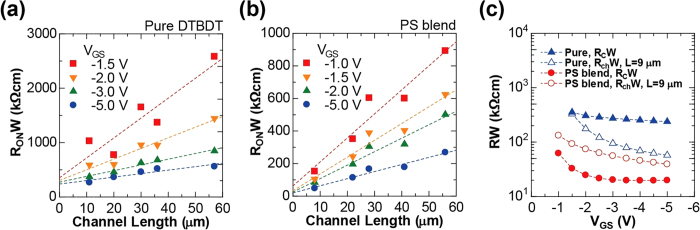
Contact resistance and channel resistance of the printed organic TFTs. Channel- width-normalized total on-resistance (*R*_ON_*W*) of (**a**) pure DTBDT-C_6_ devices and (**b**) 0.25 wt%-PS-blended DTBDT-C_6_ devices as a function of channel length and gate-source voltage (*V*_GS_). (**c**) Channel-width-normalized contact resistance (*R*_C_*W*, solid plots) and channel resistance (*R*_ch_*W*, 9 μm channel length, open plots) for pure DTBDT-C_6_ (triangle plots) and 0.25 wt%-PS-blended DTBDT-C_6_ (circle plots).

Figure 1e shows the transfer curves for ten devices with and without polystyrene (PS), respectively. The output curves were placed in Fig. S1, Supporting Information. The channel dimensions were *W*/*L* = 1070 μm/9 μm. For devices using pure DTBDT-C_6_ (dashed blue lines), the average mobility (*μ*_ave_) was 0.22 ± 0.06 cm^2^ V^−1^ s^−1^ (Maximum: 0.33 cm^2^ V^−1^ s^−1^, minimum: 0.14 cm^2^ V^−1^ s^−1^) at operating voltage of −5 V. However, for devices using DTBDT-C_6_/0.25 wt%-PS blend (solid red lines), an average mobility of 1.00 ± 0.20 cm^2^ V^−1^ s^−1^ (Maximum: 1.33 cm^2^ V^−1^ s^−1^, minimum: 0.68 cm^2^ V^−1^ s^−1^) was obtained, which is remarkably high compared to previously reported bottom-contact OTFTs using printed source/drain electrodes^20^,^21^. As a result of having employed a DTBDT-C_6_/0.25 wt%-PS blend, the statistical variation (*σ*_*μ*_/*μ*_ave_) in the standard deviation of the mobility (*σ*_*μ*_) was improved from 27% to 20% (Fig. S2, Supporting Information). The average threshold voltage (*V*_TH_) for devices processed with the DTBDT-C_6_/0.25 wt%-PS blend was 0.05 ± 0.04 V, as opposed to −0.55 ± 0.07 V for devices using pure DTBDT-C_6_. This standard deviation of the threshold voltage *V*_TH_ (0.04 V) was quite low compared with previously reported OTFT devices that were printed^15^. Figure 2f shows field-effect mobility and *V*_TH_ as a function of PS concentration. A maximum mobility of 1.0 cm^2^ V^−1^ s^−1^ was achieved for PS concentration of 0.25 wt%. As observed in polarized optical micrographs, the crystallinity of the semiconductor layer improved with increasing PS concentration up to 0.25 wt%. However, polymer concentration in excess of 0.25 wt% caused degradations in the crystallinity of the layers (Fig. S3, Supporting Information). The variations in *V*_TH_ were reduced at PS concentration of more than 0.25 wt%. Therefore, the optimum PS concentration was found to 0.25 wt%, which corresponds the condition for maximum mobility in OTFT devices using a TIPS-Pentacene/PS blend reported by X. Li *et al*.^11^.

### Extraction of contact resistance and channel resistance

To better understand the improvements in OTFT device mobility using the DTBDT-C_6_/PS blend, we extracted the channel resistance (*R*_ch_) and contact resistance (*R*_C_) from the total on-resistance (*R*_ON_) by using the transmission line method (TLM)^22^. *R*_ON_ of the OTFT device is defined as follows:


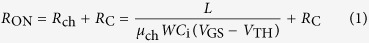


where *μ*_ch_ is the channel mobility, *C*_i_ is the insulator capacitance per unit area, and *V*_GS_ is the gate-source voltage. The channel-width-normalized *R*_ON_ were plotted as a function of channel length for the devices using pure DTBDT-C_6_ (Fig. 2a) and those using a DTBDT-C_6_/0.25 wt%-PS blend (Fig. 2b). According to Eq. 1, *R*_ch_*W* and *R*_C_*W* can be obtained from slope and y intercept of the least-squares fitted line of *R*_ON_. The extracted values *R*_ch_*W* for *L* = 9 μm and *R*_C_*W* were plotted as a function of *V*_GS_ in Fig. 2c. The *R*_C_*W* for the pure DTBDT-C_6_ devices was 238 kΩ cm at a *V*_GS_ of −5 V and the *R*_C_*W* for the DTBDT-C_6_/PS blend devices was 20 kΩ cm at a *V*_GS_ of −5 V, indicating that blending a small-molecule semiconductor and insulating polymer materials contributed to a reduction in *R*_C_. The *R*_ch_*W* of 9 μm channel length at *V*_GS_ of −5 V were also reduced from 58 to 40 kΩ cm by blending the small-molecule and polymer materials.

For discussing separately the contributions of reductions in *R*_c_ and *R*_ch_ to the improvement in effective mobility, we estimated intrinsic channel mobility (*μ*_ch_) which excludes the effect of contact resistance^23^ by using the following Equation:


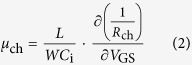


The estimated *μ*_ch_ for pure DTBDT-C_6_ and PS-blend devices was 0.56 and 0.93 cm^2^ V^−1^ s^−1^, respectively, showing that the blend of PS also improves the channel mobility by factor 1.7. The effective mobility for pure DTBDT-C_6_ devices was 2.5 times lower than the channel mobility due to the high contact resistance *R*_C_*W* of 238 kΩ cm, whereas the effective mobility for PS-blend devices was quite close to the channel mobility due to relatively low *R*_C_*W* of 20 kΩ cm, indicating that the reduction in *R*_C_*W* was attributed to get the full performance of the *μ*_ch_.

### Characterization of crystalline surface of semiconducting layer

**Figure 3:**
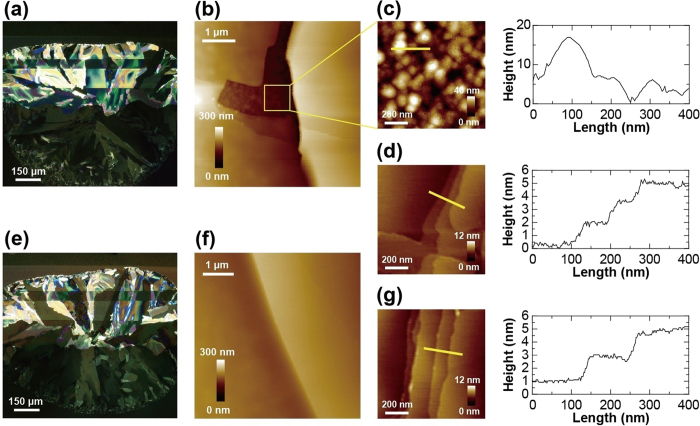
Crystalline surface of printed DTBDT-C_6_ layer. (**a**) Polarized optical micrograph of printed pure DTBDT-C_6_ crystal on parylene surface with source and drain (S/D) electrodes. (**b**) AFM image of a grain-boundary for a pure DTBDT-C_6_ channel layer. Magnified AFM image (left) and sectional profile (right) of (**c**) grain-boundary and (**d**) domains for a pure DTBDT-C_6_ channel layer. (**e**) Polarized optical micrograph of 0.25 wt%-PS-blended DTBDT-C_6_ crystal on parylene surface with S/D electrodes. (**f**) AFM image of grain-boundary of 0.25 wt%-PS-blended DTBDT-C_6_ channel. (**g**) Magnified AFM image (left) and sectional profile of a 0.25 wt%-PS-blended DTBDT-C_6_ domain.

By comparing the crystal films of OTFT devices with semiconductor layers using pure DTBDT-C_6_ and DTBDT-C_6_/PS blends, we considered the origin of reductions in channel resistance. There was no obvious difference in crystallinity of semiconducting layers such as domain size and the number of grain boundaries between pure DTBDT-C_6_ (Fig. 3a) and DTBDT-C_6_/PS blends (Fig. 3e). However, topological images evaluated using atomic force microscopy (AFM) show differences between these two semiconducting layers, whereby chasm was observed at the grain boundary in the pure DTBDT-C_6_ layer (Fig. 3b). A magnified AFM image and sectional profile of the chasm at the grain boundary (Fig. 3c) indicates a rough surface with root mean square (RMS) roughness of about 4.0 nm, which is similar to that of the parylene surface (Fig. S4, Supporting information). Therefore, there was no semiconducting layer found at the chasm region. On the other hand, these chasms were not observed and smooth connections of the domains were observed from the AFM image of DTBDT-C_6_/PS blends (Fig. 3f), which would be one of the factors of reduction in channel resistance. In magnified AFM images and sectional profiles of the pure DTBDT-C_6_ layer (Fig. 3d) and DTBDT-C_6_/PS blends domain (Fig. 3g), there was no distinct difference between two domains in which 1.8-nm-thick step and terrace surface were formed. The thickness corresponded to the intermolecular distance of DTBDT-C_6_^13^. We note that the chasms may not be the only reason why contact resistance was reduced as a result of blending. In general, contact resistance is closely related to film morphology such as chasms, energy levels, and molecular packing at the semiconductor/electrode interface^24^. We still continue studying the mechanism of reduction in contact resistance.

### Printed pseudo-CMOS inverter array with uniform electrical properties

**Figure 4:**
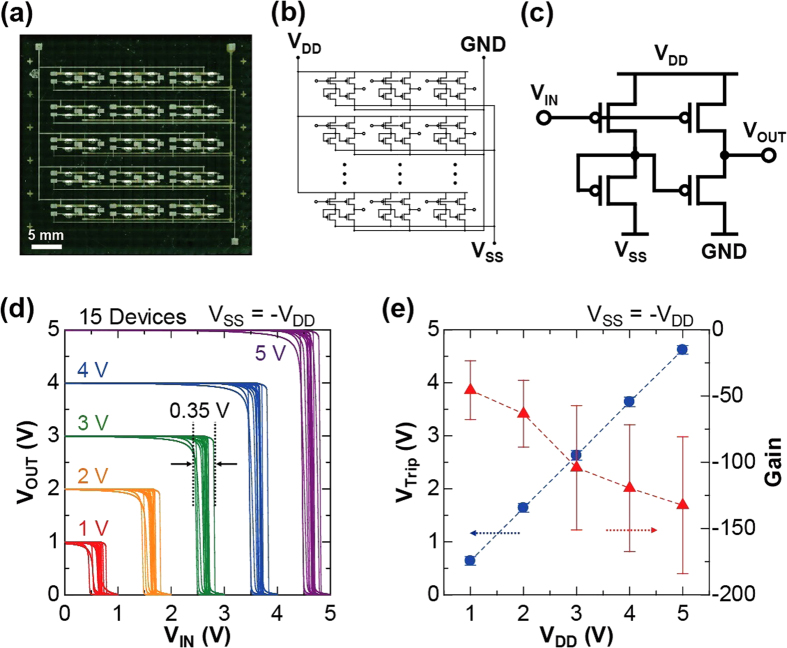
Printed pseudo-CMOS inverter array with uniform electrical performance. (**a**) Photograph of a printed organic pseudo-CMOS inverter array. Circuit diagram of the fabricated (**b**) pseudo-CMOS inverter array and (**c**) pseudo-CMOS inverter. (**d**) Static input-output characteristics of fifteen inverters in a fabricated array. The supply voltages (*V*_DD_) were set from 1 to 5 V in 1 V steps and the tuning voltage was *V*_SS_ = −*V*_DD_. (**e**) Plotted are the trip voltage (*V*_Trip_, blue solid circles) and small-signal gain (red solid triangles), as a function of *V*_DD_.

To evaluate the applicability of our DTBDT-C_6_/PS blend OTFT devices to integrated circuits, we fabricated fifteen inverters on the same substrate (Fig. 4a) whose circuit diagrams are shown in Fig. 4b. The fabricated inverters were known as the pseudo-CMOS type logic, which consists of four OTFTs for one inverter circuit (Fig. 4c)^25^. Figure 4d shows the input voltage (*V*_IN_) vs. output voltage (*V*_OUT_) characteristics of fifteen inverters with an yield of 15/15 or 100%. The estimated total variation of trip voltage (*V*_Trip_) was 0.35 V at supply voltage (*V*_DD_) ranging from 1 to 5 V. This uniformity in performance was comparable with the organic inverter circuits fabricated with evaporated semiconductor layer and metal contacts^26^, implying sufficient applicability to integrated circuits. Furthermore, all fifteen of the inverters operated at a *V*_DD_ of 1 V, which indicates that our inverter circuits were functional with low levels of power consumption. Figure 4e shows *V*_Trip_ and inverter gain (*dV*_OUT_/*dV*_IN_) plotted as a function of *V*_DD_. The standard deviation of *V*_Trip_ was 0.09 at each *V*_DD_. These results clearly suggest that the OTFT characteristics were quite uniform. As for applications, an amplifier for use in sensors^27^ could be fabricated owing to an average of inverter gain of greater than 100 at a *V*_DD_ from 3 to 5 V. Accordingly, by employing small-molecule/polymer blends, we have successfully fabricated high-performance inverters with high yield, uniform *V*_Trip_, and low-voltage operation.

## Conclusion

In summary, we have succeeded in printing short-channel OTFT devices with a high mobility, low-voltage operation and uniform electrical characteristics by employing a blend of DTBDT-C_6_ and polystyrene semiconductor materials. The small-molecule/polymer blend contributed to significant reductions in both the channel resistance (*R*_ch_) and contact resistance (*R*_C_) for printed OTFT devices with bottom-gate, bottom-contact geometries. Pseudo-CMOS inverters with excellent performance, uniformity and low-voltage operation were realized, indicating that the printed OTFT devices can be used in more complex integrated circuit applications requiring low manufacturing cost over large areas. These results will contribute to the establishment of the reliability in printed electronics.

## Methods

### OTFT Device Fabrication

Glass plates (0.7 mm thick) were used as base substrates. Cross-linked poly (4-vinyl-phenol) (PVP) material was used as a surface planarization layer. PVP (*M*_W_ ≈ 25,000, Sigma-Aldrich), and poly (melamine-co-formaldehyde) (*M*_N_ ≈ 432, 84 wt%, Sigma-Aldrich) was used as a cross-linking agent. These were mixed in propylene glycol monomethyl ether acetate. The surface of the cross-linked PVP layer was then treated for 1 min. in an oxygen plasma (plasma power of 100 W) to alter its wettability. Next, silver nanoparticle ink in an aqueous solvent (DIC Corp. Japan, JAGLT-01) was patterned as gate electrodes by using an inkjet printer (Fujifilm Dimatix, model DMP2831) with a print head having 10 pl nozzles. The silver nanoparticle ink was printed using a customized waveform and the droplets were deposited with a dot-to-dot spacing of 60 μm. During the inkjet patterning process, the substrate temperature was kept at 30 °C. After printing, the substrates were stored for 30 min. in an environmental test chamber (ESPEC, model SH-221), which was maintained at 30 °C and relative humidity of 95%RH, in order to help planarize the electrodes^18^. After the drying process, the substrates were heated at 140 °C for 30 min. to sinter the silver nanoparticles. A parylene (KISCO, diX-SR) gate dielectric layer (350 nm thick) was then formed by chemical vapor deposition. After forming the dielectric layer, the substrates were heated at 120 °C for 1 hr. in a Nitrogen atmosphere. Next, a silver nanoparticle ink in a tetradecane-based solvent (Harima Chemicals, NPS-JL) was patterned with an inkjet printer (TMP Corp. IJ-DESK) to form the source/drain (S/D) electrodes^19^. The droplets were deposited at 600 dots per inch, during which the substrate temperature was maintained at 70 °C. After the printing process, the substrates were heated at 120 °C for 1 hr. to sinter the silver nanoparticles. Next, a self-assembled monolayer (SAM) treatment for S/D electrodes was prepared by immersing the substrates in a 30 × 10^−3^ mol/L propanol solution of pentafluorobenzenethiol for 5 min. at room temperature. The substrates were then rinsed with pure propanol and dried with nitrogen. The SAM treatment changed the work function of the printed silver S/D electrodes from 4.7 to 5.4 eV, which reduces the energy barrier between the organic semiconducting layer and the S/D electrodes^28^. Fluoropolymer (DuPont, Teflon AF1600) bank layers (200 nm thick) were printed using dispenser equipment (MUSASHI Engineering, Image Master 350 PC) at a pattering speed of 20 mm s^−1^ and with a discharge pressure of 5 kPa. During the dispenser patterning process, the platen and nozzle temperatures were kept at 30 °C. After printing the bank layers, the substrates were stored in an air ambient for 10 min. to remove the solvent. The final step in the fabrication process was formation of the organic semiconducting layer, whereby a solution of DTBDT-C_6_ (1.0 wt%) and polystyrene (0, 0.1, 0.25, 0.5, 1.0 wt%, *M*_W_ ≈ 280,000, Sigma-Aldrich) blends in toluene was printed onto the area defined by the bank layers by using dispenser equipment at a patterning speed of 20 mm s^−1^ and discharge pressure of 1 kPa. During the dispenser patterning process, the platen and nozzle temperatures were maintained at 30 °C, after which the substrates were stored in an air ambient for 10 min. to remove the solvent.

## Additional Information

**How to cite this article**: Shiwaku, R. *et al*. Printed 2 V-operating organic inverter arrays employing a small-molecule/polymer blend. *Sci. Rep.* **6**, 34723; doi: 10.1038/srep34723 (2016).

## Supplementary Material

Supplementary Information
