# Supplementary material for: Printed 2 V-operating organic inverter arrays employing a small-molecule/polymer blend
Source: Sci Rep. 2016 Oct 4;6:34723. doi: 10.1038/srep34723 (PMC5048160; doi:10.1038/srep34723)
Supplement: Supplementary Information [file srep34723-s1.pdf]

Supplementary Information

Printed 2 V-operating organic inverter arrays employing a small-molecule/polymer blend

Rei Shiwaku[1], Yasunori Takeda[1], Takashi Fukuda[2], Kenjiro Fukuda[1,3], Hiroyuki Matsui[1], Daisuke Kumaki[1], Shizuo Tokito[1,a]

[1]Research Center for Organic Electronics (ROEL),
Graduate School of Science and Engineering, Yamagata University,
4-3-16 Jonan, Yonezawa, Yamagata 992-8510, Japan

[2]Functional Polymers Research Laboratory, Tosoh Corporation,
1-8, Kasumi, Yokkaichi, Mie 510-8540, Japan

[3]Japan Science and Technology Agency, PRESTO,
4-1-8, Honcho, Kawaguchi, Saitama 332-0012, Japan

[a] Author to whom correspondence should be addressed: tokito@yz.yamagata-u.ac.jp

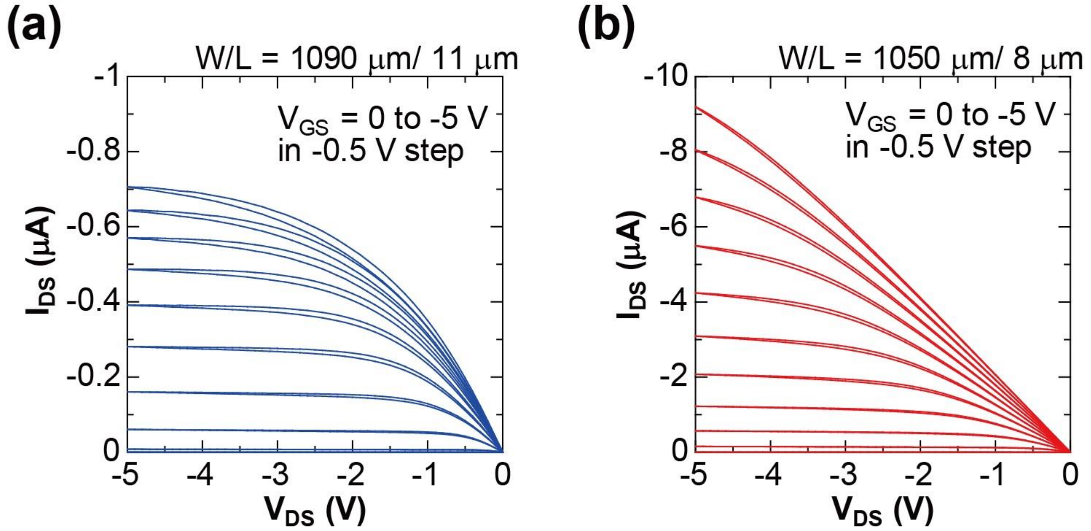

Supplementary Figure 1: Output characteristics.

V_{DS} vs. I_{DS} curves for (a) pure DTBDT-C_6 device and (b) 0.25 wt%-PS blended DTBDT-C_6 device. The output curves at low drain voltage were almost linear, indicating that the semiconductor/metal interface is Ohmic rather than Schottky.

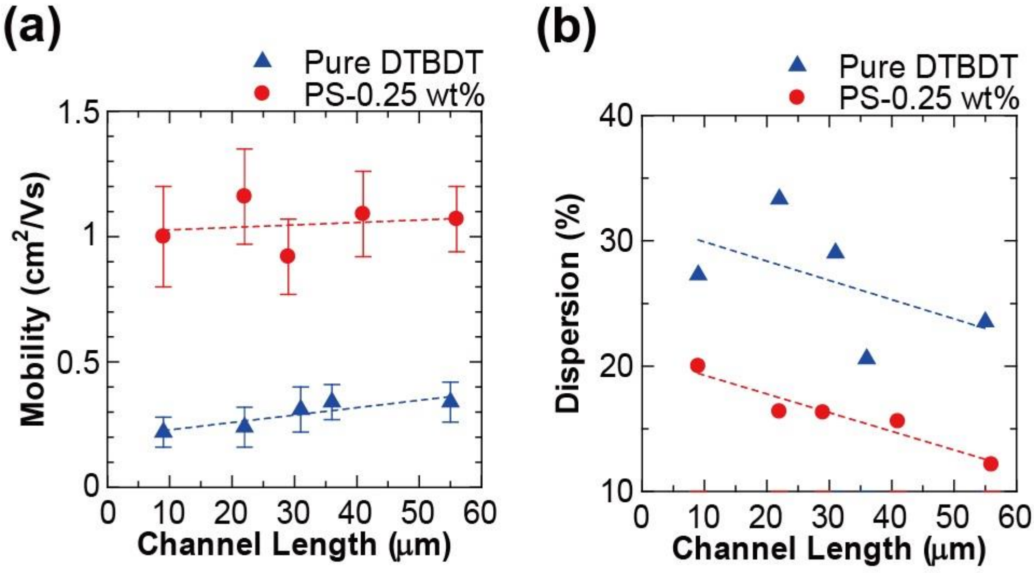

Supplementary Figure 2: Channel length dependence on mobility.

(a) Correlation between channel length and mobility. Blue triangles are for pure DTBDT-C_6 devices. Red circles are for 0.25 wt%-PS-blended DTBDT-C_6 devices. (b) Correlation between channel length and statistical dispersion (σ_μ/μ_{ave}) in mobility. Blue triangles represent pure DTBDT-C_6 devices. Red circles represent 0.25 wt%-PS-blended DTBDT-C_6 devices.

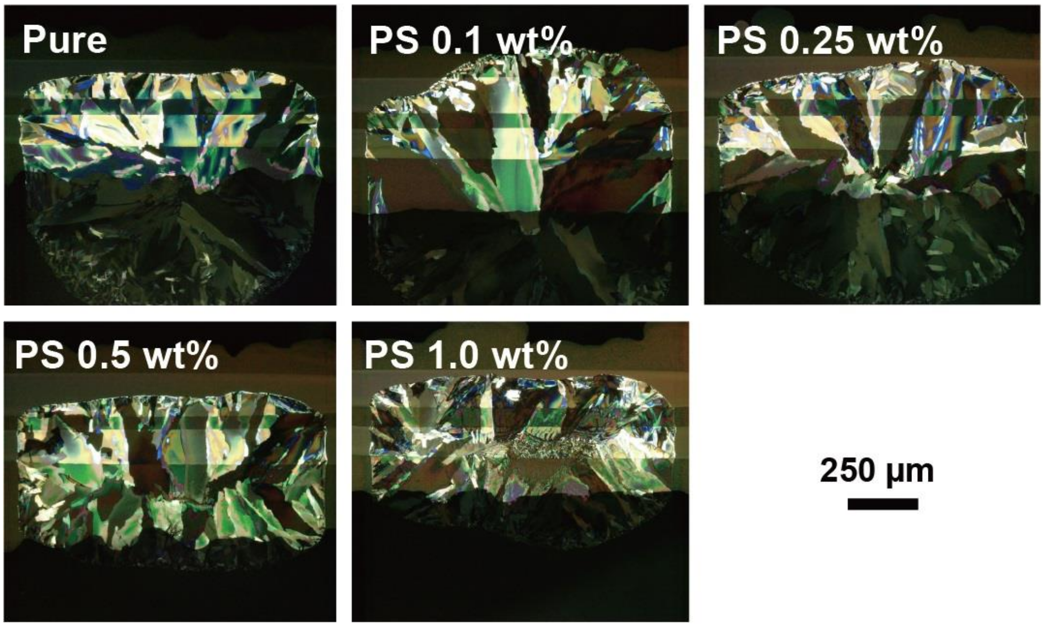

Supplementary Figure 3: Crystallinity of printed layers for DTBDT-C_6/Polystyrene blends.

Polarized optical micrograph of PS-blended DTBDT-C_6 crystalline layers with source and drain electrodes. The concentrations of blended PS were 0, 0.1, 0.25, 0.5, and 1.0 wt%. The wettability and crystalline layer quality decreased with increasing PS concentration from 0.5 wt%. 0.25 wt% was the optimum PS concentration.

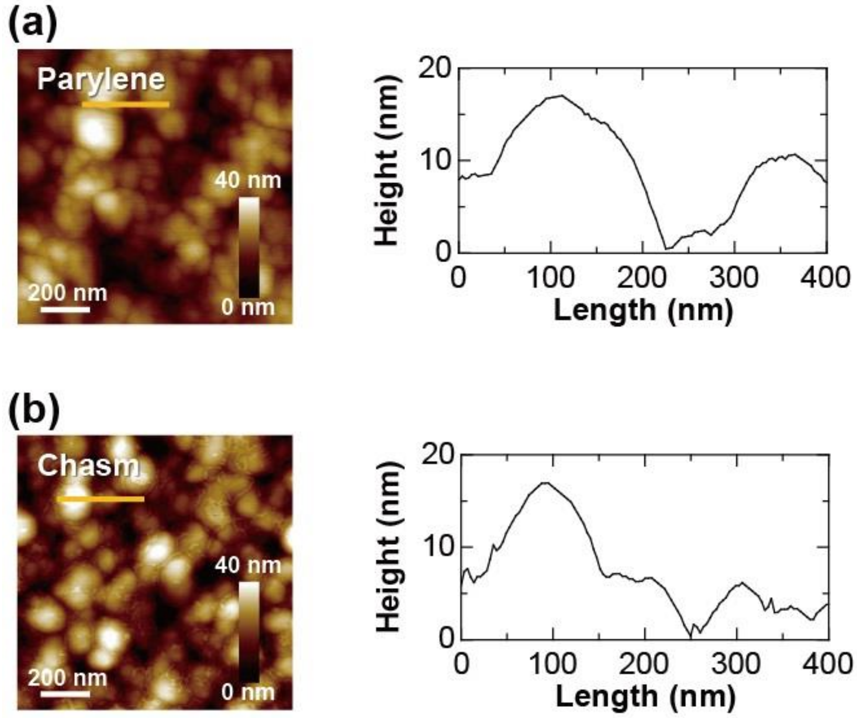

Supplementary Figure 4: AFM image and cross-sectional profile.

(a) AFM image (left) and cross-sectional profile (right) of parylene dielectric layer surface. Obtained RMS surface roughness from AFM scans was 3.45 nm. (b) AFM image (left) and cross-sectional profile (right) of a chasm in the DTBDT-C_6 crystalline surface at the channel region. Obtained RMS surface roughness from AFM scans was 4.06 nm. The grain of a chasm appears to be the parylene dielectric layer due to similarities between profiles (a) and (b).
